# Does environmental regulation improve public health? Evidence from China's Two Control Zones policy

**DOI:** 10.3389/fpubh.2023.1059890

**Published:** 2023-01-24

**Authors:** Ningze Yang, Ziwei Liu, Yuxi Lin, Yongliang Yang

**Affiliations:** ^1^School of Economics and Management, Zhejiang Sci-Tech University, Hangzhou, China; ^2^Silk and Fashion Culture Research Center of Zhejiang Province, Zhejiang Sci-Tech University, Hangzhou, China; ^3^Green and Low-Carbon Technology and Industrialization of Modern Logistics, Zhejiang Engineering Research Center, Wenzhou, China

**Keywords:** public health, Two Control Zone, policy, environmental pollution, difference-in-differences model, environmental regulation

## Abstract

Improving public health is the premise of sustainable human development and an essential condition of economic growth. However, increasing severe environmental pollution poses a threat to public health. Implementing environmental regulation policy has become a meaningful way to control environmental pollution and the basis and guarantee for achieving public health. This paper aims to study the impact of environmental regulation on public health. The Two Control Zones (TCZ) policy is the earliest and stricter environmental regulation in China. Based on the policy experiment of TCZ, this paper analyzes the role of TCZ policy in improving public health using the DID model and data from 112 cities. The study finds that the TCZ policy can significantly improve public health, and this improvement effect was continuous and lagging. The results of benchmark regression show that the implementation of the TCZ policy has reduced the incidence rate of respiratory diseases in TCZ areas by 5.7%. When considering city heterogeneity in terms of economic and geographical conditions, the study further found that the impact of improvement is largest for cities in more heavily non-provincial capital and central and western regions, respectively. In addition, the results of mediating test show that TCZ policy improves public health by reducing environmental pollution. Our research fills the gap in the literature on the micro effects of environmental regulation policy on public health in developing countries. The government should prioritize environmental pollution control through reasonable environmental regulation policies. The government should strengthen environmental information disclosure to remind the public to deal with air pollution. The government and enterprises also should take various environmental protection measures to reduce air pollution emissions.

## 1. Introduction

In the process of rapid economic development, the extensive growth mode characterized by high investment, high energy consumption, and high emissions has brought the problem of environmental pollution (1). Among them, air pollution poses a major threat to public health. Air pollution threatens human survival and leads to the rapid depreciation of healthy human capital, which also will lead to inequality in the economic development of all countries (2, 3). Therefore, governments of all countries attach importance to environmental pollution and adopt environmental laws and regulations to prevent and control environmental pollution. In 1992, the Rio Declaration issued by the United Nations pointed out that states shall enact effective environmental legislation (4). In addition, the Paris Agreement of the United Nations was signed in 2016, which shows that tackling climate change requires solid international cooperation (5). Both developed and developing countries need to take action to reduce greenhouse gas emissions and enhance their ability to cope with climate change. Implementing environmental regulation policies can promote environmental protection and reduce public health costs (6–8). However, some studies show that environmental regulation inhibits economic growth, and developing countries are reluctant to adopt strict environmental regulations. Thus, whether environmental regulation has health benefits is still a crucial question to be answered.

This paper examines the effect of environmental regulation mitigating environmental pollution on public health in China. China is one of the developing countries with rapid economic development. The extensive economic growth mode has brought about severe resource shortages and environmental pollution, which has led to the continuous deterioration of the natural living environment of residents and an increase in social health costs (9–11). The Chinese government attaches importance not only to environmental pollution issues but also to the health of residents. According to the “Report on the State of the Ecology and Environment in China 2021,” 43.1% of China's cities at and above the prefecture level failed to meet national air quality standards (12). Air pollution leads to the destruction of the ozone layer, global warming, an increase in extreme weather, and a severe impact on public health (13–16). Research shows that 81% of Chinese people live in areas with substandard air quality, and air pollution causes nearly one million deaths (17).

SO_2_ is the primary source of air pollution (18). As early as 1998, the Chinese government formulated a Two Control Zones (TCZ) policy to reduce acid rain and SO_2_ emissions. The policy aims to control acid rain and sulfur dioxide pollution, protect human health and the ecological environment, reduce economic losses, and achieve sustainable development. The TCZ policy was also the first to incorporate the environmental target assessment into the environmental regulation system. According to meteorological, topographic, soil, and other natural conditions, the policy defined areas that have been or may be polluted, including acid rain control zone and SO_2_ control zone, namely TCZ. Among them, if the pH value of rainfall is ≤ 4.5, it can be divided into an acid rain control zone; If the annual average concentration of SO_2_ in the ambient air in the past 3 years exceeds the national secondary standard, it may be designated as an SO_2_ pollution control zone (19). As a typical means of environmental regulation, the TCZ policy was formulated by the National Environmental Protection Administration and approved by the State Council. Since its implementation in 1998, it has achieved good results.

After the implementation of the TCZ policy, from 2000 to 2002, the total emission of SO_2_ in the TCZ decreased from 13.164 million tons to 11.488 million tons, with a total reduction of 1.676 million tons; National SO_2_ emissions decreased from 19.5 million tons to 19.266 million tons, with a total reduction of 685,000 tons (20). By 2010, the national SO_2_ emissions had been reduced by 14.29% compared with 2005, exceeding the binding targets of government policies (21). In particular, the national SO_2_ emissions have entered a monotonous downward trend since 2006. Urban air quality has also improved significantly.

However, the impacts of environmental regulation on public health cannot be neglected (22). The environmental regulation policies are a “win-win” situation that favors public health and economic factors (23, 24). The continuous growth of energy consumption has led to high sulfur dioxide emissions. Environmental pollution problems emerge endlessly, and public health sustainability faces enormous challenges. Therefore, the government should consider the environmental and public health relationship while formulating environmental regulation policies. We must study environmental regulation' effect on residents' health. However, previous studies' environmental results are dominated by two perspectives. Some scholars examine the relationship between environmental governance and environmental pollution (8, 9, 25, 26). Most studies focus on the impact of environmental pollution on public health (27–29). Overall, there is a lack of literature on the relationship between environmental regulation and public health and a lack of mechanism analysis of environmental pollution.

In summary, under the background of sustainable development of public health, this paper studies the path of improving public health by Implementing environmental regulation. Therefore, this paper performs an empirical analysis based on the natural experiment of China's TCZ policy and elaborates on the mechanism behind the impact of environmental regulation on public health. Using the panel data of 112 cities in China from 2004 to 2015 and the difference-in-differences (DID) method, this paper empirically analyzes the general health of pilot cities of the TCZ policy. As a typical means of environmental regulation, The TCZ policy has clearly defined the goal of phased emission reduction and governance, which is to reduce the total national emissions by 10% in 2010 compared with 2005. Therefore, this paper takes the air pollution regulation of the TCZ policy in 2010 as a natural experiment to analyze the impact of the TCZ policy on public health. The findings of this paper provide policy references for developing countries to promote public health from environmental regulation.

The possible marginal contributions of this paper are as follows. First, Changes in air quality caused by environmental regulations can solve a problem: how effective are air pollution regulations in developing countries lead to improving public health? Yang and Chou (30) examine environmental regulation on the shutdown of the Pennsylvania coal-fired power plant in the U.S. They find this regulation can improve fetal health. Tanaka (31) finds that environmental regulation can reduce the infant mortality rate based on the evidence of TCZ policy. A recent study by Liu and Zhang (32) also examines that TCZ policy can reduce annual deaths. However, our study focuses on the incidence rate of respiratory diseases. The disease incidence is a cumulative process from onset to end, which helps to make up for the lack of health effects in the mortality test. In addition, the TCZ policy focuses on applying environmental target assessment in the environmental regulation system. The findings of this paper can provide strong evidence for developing countries to establish an environmental target assessment system. Second, public health will be affected not only by environmental regulation but also by the socio-economic and regional situation. Therefore, this paper analyzes the regional heterogeneity of environmental regulation. Finally, this paper supplements the literature on the impacts of the TCZ policy in China. Previous studies focus on the policy impacts on industrial activities, enterprise innovation, foreign trade, and employment (19, 33–35). This paper expands the research mainly by studying the impact of environmental regulation on public health. We find that environmental pollution mediates the relationship between environmental regulation and public health. This paper focuses on environmental regulation and empirically analyzes the health effect of TCZ policy. This paper provides a macroscopic reference basis for understanding the impacts of environmental regulation on public health and has vital practical significance for research on public health. TCZ policy is one of the most representative environmental regulations in China. Thus, some policy suggestions for developing countries' environmental regulation and public health can be drawn based on the conclusions of this study for further sustainable development.

The structures of the paper are as follows. The second section contains the policy background and literature review. The third section introduces the model, data sources, and variable selection. The fourth section reports the empirical findings on the impact of environmental regulation on public health. The fifth section discusses dynamic effects. The sixth section analyzes the robustness test. The seventh section examines the heterogeneity. The eighth section explores the mechanism effect of environmental pollution. The ninth section provides conclusions and policy recommendations.

## 2. Policy background and literature review

### 2.1. TCZ policy

With the rapid growth of China's economy, environmental issues have attracted more and more attention from the Chinese government and society. According to the “Report on the State of the Ecology and Environment in China 1999” (36), air pollution in China was dominated by SO_2_ and soot. And the problem of acid rain is still severe. The primary source of SO_2_ is industry, accounting for 76% of total SO_2_ emissions. The acid rain area caused by sulfur dioxide-dominated air pollution accounts for about 30% of the total area and has prominent regional characteristics. The Chinese government has introduced a series of environmental control policies in response to the worsening air pollution problem. In 1998, the Chinese government issued the “Division Plan of Acid Rain Control Zone and SO_2_ Pollution Control Zone” and formally implemented the environmental regulation policy of the TCZ. TCZ policy can be divided into acid rain and SO_2_ pollution control zones. The implementation of the TCZ policy involves 175 cities in 27 provinces, and the total area of TCZ is about 1.09 million square kilometers, accounting for about 11.4% of the country's total area (37). The cities in TCZ will be subject to strict environmental control, including the use of coal, oil, and other energy, gas emissions, and the popularization of clean technologies. By 2000, the compliance rate of SO_2_ emissions of enterprises in the TCZ was 88%, and the annual sulfur dioxide emissions were reduced by 1.77 million tons (38). In 2002, the Chinese government further promulgated the 10^th^ Five-Year Plan for the Prevention and Control of Acid Rain and Sulfur Dioxide Pollution in the Two Control Zone. This policy further indicates that China will continue strengthening environmental management in the TCZ. It is worth noting that the TCZ policy establishes the target responsibility system for acid rain and SO_2_ pollution control, which is included in local officials' assessment content. The environmental targets assessment policy is essential to China's environmental regulation policy. Therefore, under the TCZ policy's background, this paper mainly examines the health effects of environmental regulation.

### 2.2. Literature review

Research on environmental pollution and public health has been a long-term concern for scholars. The government has issued a variety of environmental protection policies, especially in the case of global warming and frequent natural disasters. The existing literature on environmental regulation policies, environmental pollution, and public health can be divided into two parts. The first studies focus on the relationship between the natural environment and public health. Scholars have carried out most research on the relationship between environmental pollution and public health for a long time. Early studies introduced environmental pollution as an essential core variable in the health model (39). Further studies found that extensive economic development caused environmental pollution, especially air pollution, which directly caused much damage to human health (10, 11). Diseases caused by environmental pollution mainly include respiratory conditions, heart diseases, etc., (40, 41). Environmental pollution not only affects the health of adults but also significantly impacts children's health (42). Studies have shown that environmental pollution substantially increases perinatal mortality (43). Thus, environmental pollution reduces the labor force (44, 45) and causes economic problems. On the one hand, increasing environmental pollution does increase public health expenditures and causes income inequality (46). On the other hand, the negative impacts of environmental pollution on public health are the main ways pollution decreases general wellbeing (47–49).

Another study mainly focuses on the effect of environmental regulation on environmental pollution. Through theoretical analysis, we can see that the environment has the characteristics of public goods, and environmental pollution has negative externalities (50). Therefore, environmental regulations mainly solve the above two problems. Environmental regulations are essential for the government to provide public services to ensure general environmental needs (51, 52). Environmental regulations also restrict the economic behaviors that have external and internal diseconomies and may cause losses to public security, the environment, resources, and other interests (50, 51). Different types of environmental regulations solve environmental pollution problems in different ways. Many studies show that environmental regulation can significantly reduce PM2.5 emissions, CO_2_ emissions, and SO_2_ emissions (25, 53–55), which also impacts residents' environmental protection behavior (56) and environmental responsibility (57). At the same time, environmental regulation can improve the green production efficiency and green investment of enterprises (58). The above research verifies that formulating and implementing environmental regulation policies can effectively improve environmental quality and highlights the importance and necessity of environmental regulation.

Through the above analysis, the existing literature is rich in research on the health effects of environmental pollution and the pollution control effect of environmental regulation. However, there is a lack of research on the relationship between environmental regulation, environmental pollution, and public health. This paper focuses on the acid rain and SO_2_ control zone by China's TCZ policy. Previous studies have examined the impact of TCZ from some aspects. Such studies show that TCZ policy can reduce not only environmental pollution but also have a significant impact on industrial activities, enterprise innovation (19, 34, 59), foreign trade (33) and employment (35). The TCZ policy specifies emission levels to achieve the environmental targets of air pollution control. Therefore, this paper's novelty and archival value are to supplement the research field on the impact of environmental regulation on public health. Based on the above analysis, we propose the following research hypothesis:

H1. Relative to the public in non-TCZ areas, public health in TCZ areas was promoted by the TCZ policy.

H2. TCZ policy can promote public health by reducing the environmental pollution.

## 3. Materials and methods

### 3.1. Data sources

This paper focuses on the impact of environmental regulation on public health. In this paper, the implementation of the TCZ policy is taken as a quasi-natural experiment. The panel data of 112 cities in China from 2004 to 2015 are used as the sample. Although the explained variable has data since 2004, because the data before 2006 is seriously missing, the data before 2006 is missing when the panel data is analyzed. The environmental regulation data is obtained according to this paper's TCZ policy data measurement results (from the Division Plan of Acid Rain Control Zone and SO2 Pollution Control Zone). The other part of the data is public health, mainly from the China Cancer Registry Annual Report published by the National Cancer Center. The data in this report covered 114.1 million people from 129 urban areas. In addition to environmental regulation and public health, other indicators are from the China City Statistical Yearbook and the China Statistical Yearbook on Environment for 2005–2016. Excluding the cities with missing data, the sample size of this paper is 647.

### 3.2. Models

#### 3.2.1. Basic model

To explore the impact of environmental regulation on public health, this paper focus on the TCZ policy, which provides a quasi-natural experiment. According to Liu and Zhang (32), this paper uses DID model to conduct an empirical analysis to test whether TCZ policy can improve public health. The DID method can compare the net effect of policy intervention on TCZ and non-TCZ cities in different periods. The basic DID regression equation is set as follows:


(1) $$\begin{array}{l} PH_{it}=\alpha_{0}+\alpha_{1}TCZ_{it}+\alpha_{2}Post_{t}+\alpha_{3}TCZ_{t}\times Post_{t}+\\ \;\alpha_{4}Control_{it}+\mu_{i}+\eta_{t}+\varepsilon_{it} \end{array}$$

Where *PH*_*it*_ is public health in the city *i* in year *t*, and the residents' incidence rate of respiratory diseases is taken as a measure of public health; *TCZ*_*i*_ is the dummy variable of city grouping. If the city is in the TCZ area, *TCZ*_*i*_= 1; otherwise, *TCZ*_*i*_= 0. *Post*_*t*_ is the dummy variable of time for implementing the TCZ policy. The time point for the end of the TCZ policy is 2010. If the time is 2010 or earlier, *Post*_*t*_= 0, indicates that the TCZ policy has not been achieved; otherwise, *Post*_*t*_= 1. *TCZ*_*i*_ × *Post*_*t*_ is the product of dummy variables of TCZ and time. Therefore, this paper mainly focuses on the core coefficient α_3_, the influence coefficient of the TCZ policy on the incidence rate of respiratory diseases. In other words, the core coefficient α3 can reflect the net effect of environmental regulation on public health. *Control*_*it*_ is a set of control variables that affect public health; μ_*i*_ and η_*t*_ represent the fixed effect of city and year, respectively. ε_*it*_ is the random disturbance term.

#### 3.2.2. Dynamic model

The basic model does not reflect the dynamic effects of environmental regulation on public health. Meantime, we also should consider a time lag on the impact. Based on this, this paper conducts the dynamic development, and the equation is set as follows:


(2) $$\begin{array}{l} PH_{it}=\beta_{0}+\overset{2+}{\underset{t\geq -2}{\sum }}\beta_{t}TCZ_{i}\times Post_{t}+\beta_{1}TCZ_{i}+\beta_{2}Post_{t}+\\ \;\beta_{3}Control_{it}+\nu_{i}+\kappa_{t}+\theta_{it} \end{array}$$

*TCZ*_*i*_ × *Post*_*t*_ represents the product of TCZ policy dummy variable and year dummy variable from 2008 to 2012. The time point for the end of the TCZ policy is 2010. *Control*_*it*_ is a set of control variables that affect public health; ν_*i*_ and κ_*t*_ represent the fixed effect of city and year, respectively. θ_*it*_ is the random disturbance term. We mainly focus on the core coefficient β_t_, which is the influence coefficient of the TCZ policy on the incidence rate of respiratory diseases in the *t*th year. This treatment is equivalent to building a counterfactual parallel trend test by assuming that TCZ policy is 2 years ahead of schedule. Through the above methods, the impact of other guidelines or random factors can be excluded to accurately assess the effects of environmental regulation on public health.

#### 3.2.3. Mediation model

The existing research shows that environmental pollution seriously damages public health. Some scholars also pointed out that environmental regulation can improve environmental pollution. Therefore, to measure the mediating effect of environmental pollution on the relationship between environmental regulation and public health, we set three equations of the independent variable (TCZ policy), the intermediary variable (environmental pollution), and the dependent variable (public health) as follows:


(3) $$\begin{array}{l} PH=\delta_{1}+cTCZ+e_{1} \end{array}$$

(4) $$\begin{array}{l} EP=\delta_{2}+aTCZ+e_{2} \end{array}$$

(5) $$\begin{array}{l} PH=\delta_{3}+c^{{}^{\prime }}TCZ+bEP+e_{3} \end{array}$$

Where, *EP* is environmental pollution and *c* is the estimated coefficient of TCZ policy on public health. *a* is the estimated coefficient of TCZ policy on environmental pollution. *c*′ and *b* are the estimated coefficients of TCZ policy and environmental pollution on public health. Environmental pollution of Equation (5) is the mediating factor. According to Baron and Kenny (60) and Judd et al. (61), we test whether the coefficients *a, b*, and *c*′ are significant to determine the mediating effect of environmental pollution. Meantime, we further use Sobel to test the intermediary effect of environmental pollution (62).

### 3.3. Measures

#### 3.3.1. Public health

Public health (PH) is the dependent variable of this paper, expressed by the incidence rate of respiratory diseases. We select the incidence rate as the dependent variable that is more scientific. The disease incidence is a cumulative process from onset to death, which helps to make up for the lack of health effects in the mortality test. The respiratory diseases sources in this paper include Oral Cavity and Pharynx (but Nasopharynx, C00-C10, C12-C14), Nasopharynx (C11), Nose, sinuses, and others (C30-C31), Trachea, Bronchus and Lung (C33-C34).

#### 3.3.2. Environmental regulation

This paper's TCZ policy is the independent variable. In this paper, the achievement of the TCZ policy in 2010 is taken as a natural experiment. We judge whether 112 cities are pilot cities of the TCZ according to the Division Plan of Acid Rain Control Zone and SO2 Pollution Control Zone and further divide them into treatment groups (TCZ cities) and control groups (non-TCZ cities). Meantime, the end time of the policy 2010 is the time node to examine the impact of the realization of TCZ policy on public health. The interaction term of policy and time is a different variable, and the difference variable's regression coefficient tests the environmental regulation's health effect. The trends of the treated and control groups are shown in Supplementary Figure 1.

#### 3.3.3. Control variables

Previous medical studies show that the level of health care is the best guarantee for public health. According to the health production function of Anderson and Grossman (63), education level, income, age, gender, and other factors will affect health. Some studies also show that health is affected by different factors besides health care (64). Hence, this paper selects the control variables, including economic output level (lneco), health care (lnhea), educational level (lnedu), industrial energy intensity (lnind), air pollutants (lnAQ, lnSO_2_, lnYC), population size (lnPOP, lnbir), and meteorological factor (lnmet).

#### 3.3.4. Mediating variable

Environmental pollution (EP) is the mediating variable of this paper. This paper selects PM2.5 year-end emissions as the proxy variable of environmental pollution. The descriptive statistics of the above variables are shown in Table 1.

**Table 1:** Descriptive statistics of variables.

**Variable**	**Var-Des**	**Full Sample**		**TCZ**		**Non-TCZ**	
		**Mean**	**S.D**.	**Mean**	**S.D**.	**Mean**	**S.D**.
PH	Incidence rate of respiratory diseases (China's population standardization rate)	3.782	0.301	3.796	0.272	3.739	0.375
lnAQ	Annual mean value of PM2.5 emissions	3.619	0.495	3.649	0.416	3.529	0.672
EP	PM2.5 Discharge at the end of the year	12.913	0.794	12.892	0.815	12.980	0.726
lnhea	Number of hospitals and health centers in municipal districts	5.321	0.681	5.386	0.676	5.123	0.663
lnSO_2_	Annual emission of industrial sulfur dioxide	10.883	0.857	10.968	0.843	10.628	0.853
lnYC	Annual emission of industrial fumes	10.198	0.987	10.221	0.967	10.127	1.044
lnind	Proportion of regional industrial output value in GDP	3.906	0.194	3.894	0.201	3.940	0.169
lnedu	Educational expenditure of local finance	13.150	0.867	13.258	0.915	12.824	0.596
lnPOP	Population density	5.954	0.876	6.056	0.771	5.644	1.081
lnbir	Natural population growth rate	1.461	0.901	1.385	0.887	1.692	0.904
lnmet	Annual average relative humidity	4.189	0.114	4.203	0.109	4.147	0.120

## 4. Benchmark regression

This paper uses DID model to analyze the impact of TCZ policy on public health. Table 2 provides the estimated results of TCZ policy on public health. As mentioned above, TCZ × Post represents the interaction term between TCZ and year dummy variables; that is, it estimates the impacts of TCZ policy on public health. Column (1) indicates the impact of TCZ policy on public health after controlling regional and time-fixed effects. The results of column (1) show that implementing TCZ policy significantly negatively impacts the incidence rate of respiratory diseases at the statistical level of 5%. Columns (2) and (3) gradually add control variables based on column (1), and column (4) report the results of the bootstrap method. The results of columns (3) and (4) show that the regression coefficient is −0.216, which is statistically significant at the 1% level. Relative to the public in non-TCZ areas, public health in TCZ areas was promoted by the TCZ policy. The above regression results show that TCZ policy can significantly improve public health, which validates our hypothesis H1.

**Table 2:** Results of the benchmark regression.

**Variable**	**(1)**	**(2)**	**(3)**	**(4)**
TCZ × Post	−0.205^**^	−0.224^***^	−0.216^***^	−0.216^***^
	(−2.439)	(−2.775)	(−2.664)	(−1.485)
TCZ	0.245^***^	0.261^***^	0.258^***^	0.258^*^
	(3.100)	(3.367)	(3.316)	(1.821)
Post	0.266^***^	0.292^***^	0.291^***^	0.291^**^
	(3.477)	(3.769)	(3.762)	(2.085)
lnAQ		0.147^***^	0.148^***^	0.148^***^
		(6.634)	(6.693)	(7.800)
lnSO_2_		0.048^***^	0.049^***^	0.049^***^
		(2.645)	(2.686)	(2.743)
lnYC		−0.036^**^	−0.038^***^	−0.038^**^
		(−2.477)	(−2.589)	(−2.423)
lnhea		−0.116^***^	−0.111^***^	−0.111^***^
		(−3.993)	(−3.834)	(−4.145)
lnPOP		0.061^***^	0.062^***^	0.062^***^
		(3.054)	(3.077)	(3.346)
lnedu		−0.032	−0.030	−0.030
		(−0.748)	(−0.702)	(−0.737)
lnind		−0.054	−0.057	−0.057
		(−0.596)	(−0.629)	(−0.582)
lneco		0.041	0.042	0.042
		(1.356)	(1.368)	(1.256)
lnbir		0.028^**^	0.028^**^	0.028^**^
		(2.126)	(2.101)	(2.207)
lnmet			−0.102	−0.102
			(−0.964)	(−0.974)
Constant	3.502^***^	1.696^***^	2.061^***^	2.061^***^
	(48.485)	(3.515)	(3.308)	(3.106)
City fixed effect	YES	YES	YES	YES
Year fixed effect	YES	YES	YES	YES
Observations	647	641	640	640
R-square	0.029	0.142	0.144	0.144
Number of areas	112	112	112	112

^*^p < 0.10, ^**^p < 0.05, ^***^p < 0.01. t-statistics in parentheses.

The results of columns (3) and (4) show 5.7% [(0.216/3.782) ^*^100% = 5.7%] reductions in the incidence rate of respiratory diseases over the 6 years in the post-policy period. Compared with previous studies, Tanaka (31) finds that the infant mortality rate fell by 20% in the TCZ areas. In addition, Liu and Zhang (32) find that the implementation of the TCZ policy has reduced the mortality rate (including adults) in TCZ areas by 5.2%. Our study also further verifies the conclusions of previous studies. It is worth mentioning that TCZ policy is an environmental regulation to control air pollution. Mainstream studies show that air pollution is the leading cause of respiratory and heart disease (40, 41). We use the incidence rate of respiratory diseases instead of the mortality rate to examine the level of public health, which can better obtain the health effect of environmental regulation. Overall, our study finds that the strict implementation of the TCZ policy can significantly promote public health.

## 5. Dynamic effects analysis

The DID model should meet the parallel trend assumption, which is no significant difference between the treatment and control groups before implementing the policy. In addition, the benchmark regression results have shown that the coefficient has reflected the average impact of TCZ policy on public health but not the dynamic impact in different periods. Hence, considering the time lag effect of TCZ policy, according to equation (2), we examine the dynamic effects of TCZ policy on public health each year, as shown in Table 3.

**Table 3:** Results of dynamic effects.

**Variable**	**(1)**	**(2)**	**(3)**
TCZ × Post_2008_	−0.111	−0.124	−0.124
	(−1.333)	(−1.523)	(−1.516)
TCZ × Post_2009_	−0.108	−0.102	−0.102
	(−1.364)	(−1.270)	(−1.270)
TCZ × Post_2010_	−0.151^**^	−0.163^**^	−0.161^**^
	(−2.101)	(−2.325)	(−2.272)
TCZ × Post_2011_	−0.156^**^	−0.169^**^	−0.166^**^
	(−2.173)	(−2.463)	(−2.399)
TCZ × Post_2012_	−0.162^**^	−0.175^***^	−0.173^***^
	(−2.446)	(−2.733)	(−2.673)
Control variables	No	Yes	Yes
City fixed effect	Yes	Yes	Yes
Year fixed effect	Yes	Yes	Yes
Observations	647	645	646
R-square	0.059	0.103	0.101
Number of areas	112	112	112

^**^p < 0.05, ^***^p < 0.01. t-statistics in parentheses. Column (2) does not contain meteorological factor variables.

The six variables in Table 3, TCZ × Post2008 to TCZ × Post2012, correspond to the impact of TCZ policy on public health in the first through sixth years. Table 3 shows that the regression coefficients of TCZ × Post2008 and TCZ × Post2009 are insignificant, indicating no difference between the treatment group and the control group in 2008–2009, which conforms to the parallel trend assumption. The coefficients of TCZ × Post_2010_, TCZ × Post_2011_, and TCZ × Post_2012_, are significantly negative, indicating that public health in TCZ areas has become better than that of public health of non-TCZ from 2010. In addition, the TCZ policy has long-term effects on public health, meaning that the impact of the implementation of environmental regulation on public health has a lag effect, and the result is gradually increasing. Whether health damage or health recovery is a long-term accumulation process (40, 41), the reduction or growth of the current incidence may also be the cumulative result of environmental pollution or improvement in the past few years. This accumulation process reflects that there may be a certain lag in the health effect of environmental policies. The reason for the gradual increase in the health effect of the policy may be that the “12^th^ Five-Year Plan” has opened a new round of environmental governance in China. With the passage of this period, environmental regulation has been strengthened, and the effect of environmental regulation has become increasingly prominent. Therefore, the effects of environmental regulation will be significantly enhanced with the promotion of environmental policies.

## 6. Robustness test

This paper uses three robustness test methods. Firstly, the China Cancer Registry Annual Report data from 2004 to 2006 were missing, so the sample window should be adjusted. We exclude the data from 2004 to 2006 and only retain the data from 2007 to 2015 for regression analysis. Column (1), (2), and (3) of Table 4 reports the results of the adjusted sample window. Column (1) does not add control variables. Columns (2) and (3) gradually add control variables based on column (1). We find that TCZ policy significantly improves public health, indicating that the results are robust.

**Table 4:** Results of robustness test.

**Variable**	**(1)**	**(2)**	**(3)**	**(4)**	**(5)**	**(6)**	**(7)**	**(8)**
TCZ × Post	−0.205^**^	−0.224^***^	−0.216^***^	−0.223^**^	−0.248^***^	−0.239^***^	−0.238^*^	−0.235^*^
	(−2.439)	(−2.775)	(−2.664)	(−2.466)	(−2.879)	(−2.770)	(−1.672)	(−1.695)
TCZ	0.245^***^	0.261^***^	0.258^***^	0.267^***^	0.285^***^	0.283^***^	0.274^*^	0.279^**^
	(3.100)	(3.367)	(3.316)	(3.139)	(3.444)	(3.410)	(1.928)	(2.067)
Post	0.266^***^	0.292^***^	0.291^***^	0.282^***^	0.289^***^	0.290^***^	0.312^**^	0.283^**^
	(3.477)	(3.769)	(3.762)	(3.501)	(3.576)	(3.586)	(2.238)	(2.064)
Constant	3.502^***^	1.696^***^	2.061^***^	3.485^***^	1.809^***^	2.318^***^	1.540^***^	1.484^***^
	(48.485)	(3.515)	(3.308)	(45.523)	(3.143)	(3.072)	(3.411)	(3.440)
Control variables	No	Yes	Yes	No	Yes	Yes	Yes	Yes
City fixed effect	Yes	Yes	Yes	Yes	Yes	Yes	Yes	Yes
Year fixed effect	Yes	Yes	Yes	Yes	Yes	Yes	Yes	Yes
Observations	647	641	640	589	584	583	646	646
R-square	0.029	0.142	0.144	0.032	0.161	0.163	0.140	0.128
Number of areas	112	112	112	104	104	104	112	112

^*^p < 0.10, ^**^p < 0.05, ^***^p < 0.01. t-statistics in parentheses. Columns (2) and (5) do not contain meteorological factor variables.

Secondly, the Chinese government has taken tremendous air pollution control measures to improve air quality and control air pollution in preparing for the 2008 “Green Olympics.” As a result, the pollution regulations are increased in the short term, and the six major cities of the Olympic Games, namely Beijing, Hongkong, Qingdao, Shanghai, Shenyang, and Tianjin, are excluded. In addition, the economic situation of the first-tier cities is better than that of ordinary towns. There are differences in financial expenditure for environmental governance, which may have endogenous problems. Therefore, Shenzhen and Guangzhou are also excluded. We will further conduct an empirical analysis of the remaining 104 cities. Columns (4), (5), and (6) of Table 4 report the results of the reselecting sample. Column (4) does not add control variables. Columns (5) and (6) gradually add control variables based on column (4). Column (6) results show that the regression coefficient is −0.239, which is statistically significant at the 1% level. After reselecting the sample, the results show that the TCZ policy can significantly improve public health.

Thirdly, we combined the DID with the PSM (Propensity Score Matching) method for the robustness test. We use the probit model and logit to estimate the propensity score. The probability density of the propensity score is shown in Supplementary Figure 2. Then, based on the propensity score above, the TCZ and non-TCZ cities are matched. We adopt the K-nearest neighbor matching (k = 1) method. Columns (7) and (8) of Table 4 report the results of the PSM-DID. The coefficients of TCZ × Post in columns (7) and (8) are negative and statistically significant at the 1% level. The above results are consistent with the benchmark regression results. In summary, this paper considers that the TCZ policy plays an important role in reducing sulfur dioxide and SO_2_ emissions and improving public health.

## 7. Heterogeneity analysis

The economic and geographical conditions of Chinese cities are highly heterogeneous, and the phenomenon of regional economic development differentiation still exists. Therefore, this paper divides the sample areas into provincial capital cities and non-provincial ones. The impact of regional economic diversity on public health in China is different. The effect of environmental regulation may be different due to other economic development conditions in the eastern regions and central and western regions. Therefore, according to the geographical characteristics, the samples are divided into eastern regions and west and central regions. Table 5 presents the results.

**Table 5:** Results of heterogeneity analysis.

**Variable**	**(1)**	**(2)**	**(3)**	**(4)**
	**Provincial capital**	**Non-provincial capital**	**Eastern regions**	**Central and western regions**
TCZ × Post	−0.050^**^	−0.101^***^	−0.014	−0.114^***^
	(2.186)	(−2.784)	(−0.214)	(−2.828)
Contant	3.853^***^	5.690^***^	1.379	6.017^***^
	(3.438)	(6.413)	(0.695)	(6.373)
Control variables	Yes	Yes	Yes	Yes
City fixed effect	Yes	Yes	Yes	Yes
Year fixed effect	Yes	Yes	Yes	Yes
Observations	73	568	282	359
R-squared	0.351	0.125	0.170	0.147
Number of areas	10	102	46	66

^**^p < 0.05, ^***^p < 0.01. t-statistics in parentheses.

As shown in columns (1) and (2) of Table 5, the regression coefficient of TCZ policy in provincial and non-provincial capital are −0.050 and −0.101, which are statistically significant at the 5 and 1% levels. It can be seen from the comparison that the implementation of environmental regulation has a more substantial effect on the improvement of public health in non-provincial capital cities. In addition, as shown in columns (3) and (4) of Table 5, TCZ policy only significantly negatively impacts public health in central and western regions. On the one hand, the medical level in economically developed areas is higher than that in economically underdeveloped regions, leading to a higher level of public health in economically developed areas. Therefore, the health effects of environmental regulation are not evident in economically developed areas. On the other hand, the higher level of industrial technology in economically developed areas can reduce environmental pollution to a greater extent, thereby reducing public health damage. Therefore, the health effect of environmental regulation in economically developed areas is not apparent. It is worth noting that the role of TCZ policy on public health in the eastern region is not significant, which also allows us further to study the way and intensity of environmental pollution control to provide research direction.

## 8. Mechanism analysis

The above empirical results have shown that environmental regulation significantly improves public health. We further discuss the environmental pollution in which TCZ policy affects public health. Table 6 presents the results of the mechanism analysis. As shown in column (1) of Table 6, the TCZ policy still significantly improves public health. As shown in columns (2) and (3) of Table 6, the results of regression coefficients (*a, b, c*′) show that TCZ policy significantly reduces environmental pollution, and TCZ policy and pollution significantly improve and damage public health, respectively. The mechanism effect accounted for 35.4% of the total effect. The above results indicate that the reduction of environmental pollution plays a part in the plausible channel in the path of TCZ policy to improve public health, which validates our hypothesis H2.

**Table 6:** Results of mechanism analysis.

**Variable**	**(1)**	**(2)**	**(3)**
	**Path *c***	**Path *a***	**Path *b* and *c^′^***
TCZ	−0.184^**^	0.209^*^	−0.220^***^
	(−2.216)	(1.916)	(−2.713)
EP			0.173^***^
			(5.836)
Constant	3.214^***^	7.302^***^	1.950^***^
	(5.301)	(9.152)	(3.100)
Control variables	Yes	Yes	YES
Sobel test	−0.017^***^		
Proportion of indirect effects	35.4%		
Observations	640	640	640
R-squared	0.099	0.775	0.146
Number of areas	112	112	112

^*^p < 0.10, ^**^p < 0.05, ^***^p < 0.01. t-statistics in parentheses.

## 9. Conclusion and recommendations

Environmental regulation reducing environmental pollution is critical to improving public health. Our study provides empirical evidence of environmental regulations improving public health in developing countries. Based on panel data from 112 cities for 2006–2015 and a quasi-experimental of TCZ policy, this paper discusses the impact of environmental regulation on public health. According to previous studies, environmental regulation can effectively improve environmental quality (25) and promote the green investment of enterprises (58). In addition, Mainstream research shows that environmental pollution is the main factor leading to the public health crisis (40, 41). However, there is a lack of literature on public health from environmental regulation perspective. We find that the TCZ policy can significantly improve public health (5.7% reductions in the incidence rate of respiratory diseases), and this effect is continuous and lagging. Further heterogeneity analysis, the TCZ policy has a more significant effect on public health for provincial capitals and central and western cities. After conducting the robustness test from three methods, the TCZ policy still significantly improves public health. The results of the mechanism analysis show that the TCZ policy improves not only public health but also public health by reducing the environmental pollution. This paper focuses on China's TCZ policy and expounds on the impact of environmental regulation on public health improvement. It provides an empirical basis for understanding the effect of environmental regulation on public health improvement and has important practical significance for public health research.

These findings in this paper have vital policy implications for improving developing countries' public health from the perspective of environmental regulation realization. The government should improve environmental laws and regulations and formulate reasonable environmental policies. During the implementation of the policy of TCZ, good environmental governance results have been achieved. The procedure is adapted to local conditions and requires different pollutant emissions in other regions. Therefore, the government should comprehensively consider the space-time distribution of pollution sources and regional economic development and formulate reasonable environmental targets. All regions must continue to promote environmental protection and the development of public health. The government should improve public health for underdeveloped regions by implementing stricter environmental regulation policies. On the other hand, environmental regulation should aim not only at pollution control but also to improve public health. Developing countries can adopt target-related responsibility systems in environmental protection. The responsibility system is an administrative system that explicitly implements the responsibility of local governments at all levels and pollution-related units for environmental quality. The government should pay more attention to the damage to public health caused by environmental pollution. At the same time, the government should strengthen environmental information disclosure to remind the public to deal with air pollution. The government and enterprises also should take various environmental protection measures to reduce air pollution emissions. In addition, the government should fully mobilize residents to participate in environmental protection, increase the public's attention to air pollution, and continue to improve medical and health services. Reasonable environmental and medical policies can effectively reduce the incidence of respiratory diseases and the health hazards and medical losses caused by environmental pollution.

This study complements the lack of relevant research on environmental regulation and public health and provides clear policy implications for developing countries. However, we note that this study's limitation needs to be further improved. First, the impact of environmental regulation on different types of diseases is different. In addition, we also lack data on different diseases. Future studies should expand in data richness and identify the health effects of environmental regulation policies in different periods. Second, we are prepared to discuss different mechanisms (such as the regulatory role of local government competitiveness and the mediation role of enterprise green innovation) of the relationship between environmental regulation and public health in future research. Finally, the research on public health should not only focus on the macro level of environmental regulation policy but also explore the micro level, such as the influence of residents' cognition of environmental protection policy on environmental protection behavior, health effects of residents' environmental behavior, and residents' mental health. Therefore, we plan to design questionnaires and conduct field surveys to expand the research on residents' health (physical and mental health) based on microdata.

## Data availability statement

The data analyzed in this study is subject to the following licenses/restrictions: The data are available from the corresponding author upon reasonable request. Requests to access these datasets should be directed to royyang@zju.edu.cn.

## Author contributions

NY: literature review, methodology, and revision. ZL: empirical analysis and software. YL: literature collection and data curation. YY: conceptualization, critical review, and supervision. All authors contributed to the article and approved the submitted version.
