# Supplementary figures and images for: Does environmental regulation improve public health? Evidence from China's Two Control Zones policy
Source: Front Public Health. 2023 Jan 24;11:1059890. doi: 10.3389/fpubh.2023.1059890 (PMC9902943; doi:10.3389/fpubh.2023.1059890)

**Supplementary Figure 1.** The trends of the treated and control group

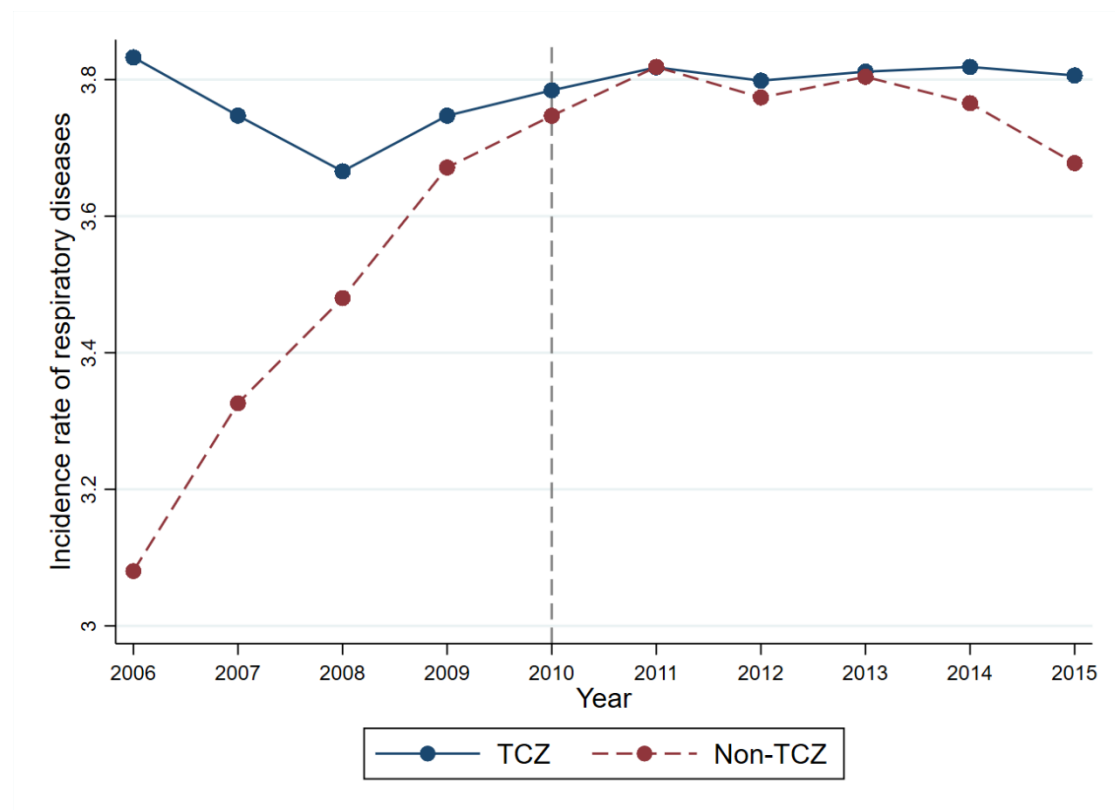

Supplement: Supplementary file 1 [file Data_Sheet_1.PDF]

**Supplementary Figure 2.** The probability density of the propensity score

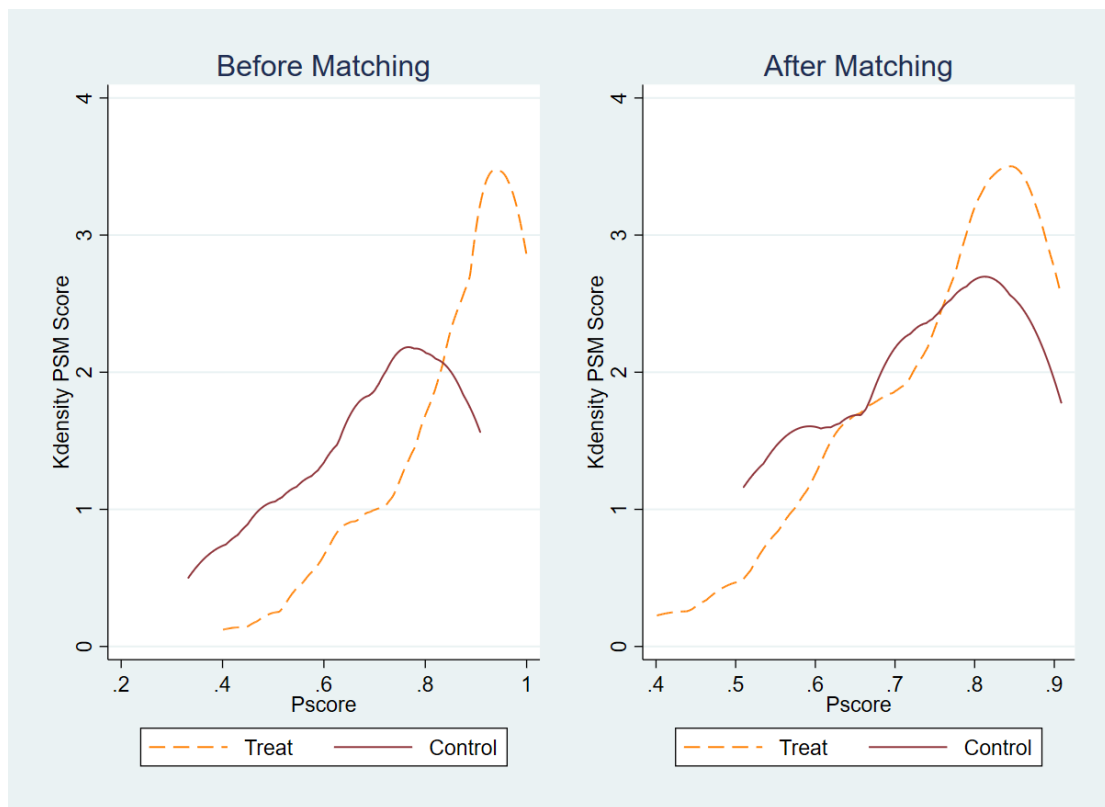

Supplement: Supplementary file 2 [file Data_Sheet_2.PDF]
